# Cellulose Isolated From Waste Rubber Wood and Its Application in PLA Based Composite Films

**DOI:** 10.3389/fbioe.2021.666399

**Published:** 2021-03-31

**Authors:** Zhiqiang Ou, Qi Zhou, Xin Rao, Haifeng Yang, Chunqing Huo, Xueyu Du

**Affiliations:** ^1^Hainan Provincial Fine Chemical Engineering Research Center, Hainan University, Haikou, China; ^2^Hainan Provincial Key Laboratory of Fine Chemicals, Hainan University, Haikou, China; ^3^School of Materials Science and Engineering, Hainan University, Haikou, China

**Keywords:** rubber wood cellulose, cellulose nanocrystals, acetylation, polylactic acid, composite film

## Abstract

Waste rubber wood (RW) is the castoff of rubber plantation with abundant reservation but without high-value utilization. In this study, cellulose with high purity has been efficiently isolated from waste RW and further processed into cellulose nanocrystals. By means of acetylation, more hydrophobic cellulose-based products, namely acetylated rubber wood cellulose (Ac–RWC) and acetylated rubber wood cellulose nanocrystals (Ac–RW–CNC) had been attempted as reinforcing fillers for fabricating two series of PLA-based composite films *via* spin coating instead of currently prevailing melt compounding technique. To ensure a uniformed dispersion of fillers in PLA matrix, the addition of reinforcing filler should be equal to or less than 5% based on the film dry weight. Compared with pure PLA film, the Ac–RWC reinforced PLA composite films are more thermally stable, while the Ac–RW–CNC reinforced PLA composite films on the other hand exhibit more enhanced performance in mechanical properties and the degree of crystallinity. The highest tensile strength (55.0 MPa) and Young’s modulus (3.9 GPa) were achieved for 5%Ac–RW–CNC/PLA composite film.

## Introduction

Under the requirements of environment protection and sustainable development, large-scale limitation or complete prohibition of disposable plastic products has been implemented by an increasing number of countries and regions (Niu et al., 2018). As promising alternatives of fossil-based plastics, polylactic acid (PLA) along with other well-known biodegradable polymers (e.g., polyvinyl alcohol, polycaprolactone, polyhydroxybutyrate, etc.) have attracted extensive attention in recent years (Chen et al., 2003; Avinc and Khoddami, 2010; Abdulkhani et al., 2014; Niu et al., 2018; Mosnackova et al., 2020). PLA is defined as a linear aliphatic polyester that is mainly manufactured by ring opening polymerization of lactide in industry. Although numerous merits (e.g., non-toxicity, non-irritation, biodegradability, good processability, comparable mechanical properties, etc.) guarantee its critical status both in academia and industry (Abdulkhani et al., 2014; Murariu and Dubois, 2016; Santoro et al., 2016; Tajbakhsh and Hajiali, 2017; Niu et al., 2018), poor thermal stability, high brittleness, and limited gas barrier property are still the limiting factors of PLA in packaging field (Abdulkhani et al., 2014; Liu et al., 2014).

To further remedy the property-limitation mentioned above, blending PLA matrix with natural polymers *via* either melt compounding or solvent casting has been adopted as the feasible pathways and many efforts have been carried out accordingly (Oksman et al., 2006; Bondeson and Oksman, 2007; Jonoobi et al., 2012). The characteristics of natural polymers (e.g., cellulose, starch, etc.) not only meet the basic requirements of green and sustainable chemistry (e.g., biodegradability, renewability, and cost-effectiveness), but would also endow the ultimate PLA-based bio-composites with improved mechanical properties, thermal stability, and flexibility (Kamal and Khoshkava, 2015; Niu et al., 2018). To date, numerous species of lignocellulosic wastes (e.g., bagasse, cornstalk, coconut husk, etc.) have been attempted for isolation of cellulose (Ditzel et al., 2017; Tuerxun et al., 2019; Wu et al., 2019; Ejaz et al., 2020; Kian et al., 2020; Wang H. et al., 2020) and meanwhile the obtained cellulose could be further processed for high-value utilization.

As a high value-added product disintegrated from cellulose, cellulose nanocrystals (CNCs) possess low density, high crystallinity, and superior stiffness, which are therefore can be employed as a highly promising reinforcement filler for PLA-based films (Ditzel et al., 2017; Tuerxun et al., 2019; Kian et al., 2020). However, the hydrophilic CNCs are theoretically difficult to be uniformly dispersed within the hydrophobic matrix of PLA, for their limited interfacial compatibility (Sullivan et al., 2015; Dong et al., 2017). Proper surface modifications *via* acetylation or silylation are thus motivated to convert hydrophilic hydroxyls of CNC into more hydrophobic functional groups (Kamal and Khoshkava, 2015). In this manner, the partial loss of thermal stability from high crystalline CNC would be timely remedied by the reinforced interfacial interactions between acetylated CNCs and PLA molecular chains.

Rubber tree is one type of important industrial plant resources, which plays a significant role in communication and military industries (Li et al., 2013). Hainan is the largest production base of natural rubber in China and annually a vast amount of rubber wood (RW) wastes from both rubber plantation and rubber processing industry has been generated without efficient utilization. Therefore, rational development of this type of cellulose-rich feedstock not only mitigates the atmospheric pollution caused by conventional incineration of lignocellulosic wastes, but also excavates the intrinsic value of natural polymers present in biomass wastes for conversion of high value-added products (Abdulkhani et al., 2014; Wu et al., 2019; Wang H. et al., 2020).

The aim of the present study is to realize the high value utilization of rubber wood cellulose (RWC) by developing two binary bio-composite film systems composed of acetylated cellulose-based filler and PLA matrix. Moreover, spin coating technique was employed to overcome the drawbacks such as low dispersibility and high energy cost brought about by solvent casting and melt compounding, respectively. Meanwhile, whether the acetylated cellulose-based filler would eventually strengthen interfacial interactions with hydrophobic PLA molecular chains during the process of spin coating will be evaluated. On the basis of comprehensive characterization of various RWC-based products by means of FTIR, XRD, SEM, TEM, and TGA, two sets of PLA–based composite films had been designed, denoted as acetylated rubber wood cellulose reinforced PLA (Ac–RWC/PLA) composite films and acetylated rubber wood cellulose nanocrystal reinforced PLA (Ac–RW–CNC/PLA) composite films. The performance of different film products had been further evaluated and compared in term of their mechanical properties, surface morphology, thermal stability, crystallization behavior, and light transparency.

## Materials and Methods

### Materials

Rubber wood (RW) waste was collected from a local rubber plantation in Hainan, China. Commercial polylactic acid (Ingeo 4032D) with density of 1.24 g/cm^3^ was provided by Yeqiang Plastic Materials Co., Ltd. (Dongguan, China). Sodium chlorite, sodium hydroxide, and acetic acid were purchased from Aladdin Co., Ltd. Acetic anhydride was supplied from Guangzhou Chemical Reagent Co., Ltd. Benzene, ethanol, and other chemicals were provided by Macklin Co., Ltd. Deionized water was used for all the experiments.

### Separation and Purification of Rubber Wood Cellulose

**FIGURE 1:**
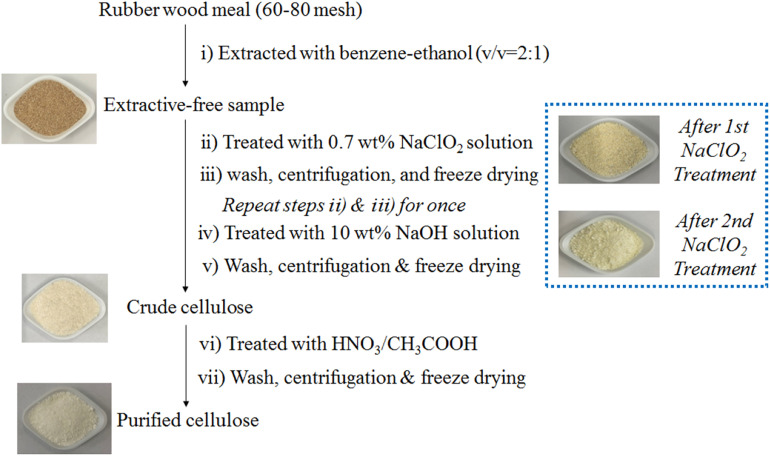
The technical flowchart of separation and purification of rubber wood cellulose.

Rubber wood cellulose with high purity was isolated following successive treatments of extractives removal, delignification, and residual lignin removal. The entire technical flowchart was presented in Figure 1. In brief, the clean and pre-cut RW chips (around 0.4 cm × 0.8 cm) were disintegrated into the size of 60∼80 mesh by using a FZ102 plant mini-mill (Taisite Instrument Ltd., China). Then the milled wood meal was extracted with benzene-ethanol (v/v = 2:1) for 24 h by using a Soxhlet extractor for thorough removal of extractives. Charge given amounts of extractive-free sample and 0.7 wt% sodium chlorite aqueous solution into a flask equipped with a reflux condenser, and mix the suspension under magnetic stirring. The solid-liquid ratio was set as 1:60 and the pH value of the suspension was adjusted to four by addition of 10 wt% acetic acid aqueous solution. The subsequent sodium chlorite (0.7 wt%) treatment proceeded under 85°C for 120 min and the residue was thoroughly washed with deionized water and centrifugated prior to a repeated run of the above sodium chlorite delignification step. After a further extraction step with 10 wt% sodium hydroxide aqueous solution, the crude cellulose was reclaimed by deionized water rinsing, centrifugation, and freeze drying. The ultimate purified cellulose was acquired by means of a core treatment step consisting of 68 wt% nitric acid aqueous solution and 80 wt% acetic acid aqueous solution as well as the following impurity removal and freezedrying. The related operating parameters are listed as follows: V_68 *wt% HNO3*_:V_80 *wt% HNO3*_ is 1:9; sample–mixed acids solution ratio is 1:20; treatment temperature is 120°C; treatment time is 15 min.

### Preparation of Rubber Wood Cellulose Nanocrystals

One gram purified RWC and 10 mL 64 wt% sulfuric acid solution were introduced into a 50 mL conical flask sealed with a glass stopper. The acid hydrolysis was carried out at 50°C for 1 h under magnetic stirring and the reaction was quenched by dilution of excess deionized water. After thorough rinsing under vacuum filtration, the wet cake together with 50 mL deionized water was transferred into a double-layered glass cup that was connected with a recirculating cooler. Once the temperature cooled down to 4°C, the slurry was intermittently treated by a JY98-IIIL sonicator (Dekelaier Instrument Ltd., China) under the power of 1,200 W. A single cycle of sonication (5 s) includes 1 s of sonication and 4 s of pause, and the accumulated sonification time is 20 min. The rubber wood cellulose nanocrystals (RW-CNC) were then reclaimed by dialysis and freeze drying of the resultant slurry and kept in a desiccator for future use.

### Acetylation of RWC and RW-CNC

Pre-submerge 2 g RWC in 50 mL acetic acid at 50°C under magnetic stirring for 30 min. Then charge 10 mL acetic anhydride and 10 μL 98 wt% sulfuric acid into the suspension and keep the reaction under 90°C for 180 min with continuous stirring. When the time was due, quench the reaction with 75% ethanol and collect the precipitate by vacuum filtration. After thorough rinse with deionized water and lyophilization, acetylated RWC (Ac–RWC) was reclaimed and kept in a desiccator for future use. The procedure for preparation of acetylated RW–CNC (Ac–RW–CNC) was similar as the case of acetylation of RWC except a smaller amount (0.5 g) of the starting material.

### Chemical Composition Analysis

The contents of extractives, ash, Klason lignin, and acid soluble lignin were determined according to TAPPI standard methods, namely TAPPI T204 cm-97, TAPPI T211 om-07, TAPPI T222 om-06, and TAPPI UM 250. The carbohydrate analysis was carried out following the standard procedure of TAPPI T222 om-02. Each measurement was performed in duplicate.

### Preparation of the Composite Films

Fully dissolve a given amount of Ac–RWC or Ac–RW–CNC in a PLA containing dichloromethane solution and set the obtained solution still for a while to remove residual air bubbles. The composite films were fabricated by using a KW-4A spin coater (Institute of Microelectronics of the Chinese Academy of Sciences, China). Briefly, the solution was dropped slowly onto a clean square polyethylene substrate. The initial spin speed was set as 600 rpm for 6 s, and then followed by 3,000 rpm for 40 s. Detach the substrate and place it horizontally in hood for 48 h to fully evaporate dichloromethane. Peel the film from the substrate and keep it in a plastic bag for future analysis.

### Characterization

#### Fourier Transform Infrared Spectroscopy

The FTIR spectra of five different fiber samples, denoted as RW, RWC, RW–CNC, Ac–RWC, and Ac–RW–CNC were illustrated by applying a Bruker Tensor 27 FTIR spectrometer. Each spectrum was recorded from 4,000 to 500 cm^–1^ with 32 scans at a resolution of 4 cm^–1^.

#### X-ray Diffraction Analysis

Five different fiber samples were analyzed by using a Rigaku Smart Lab X-ray diffractometer equipped with a Ni-filtered CuKα radiation at 30 mA and 40 kV. Each XRD pattern was plotted from 5° to 40° and the scanning rate was 5°/min. The sample crystallinity was estimated according to the method established by Segal et al. (1959).

#### Determination of Zeta Potential

Zeta potential of RW–CNC dispersed in its sonicated suspension was characterized by using a Zetasizer Nano ZS90 (Malvern Instruments Ltd., United Kingdom). Each measurement was performed in triplicate.

#### Morphology Characterization

A Phenom ProX scanning electron microscopy (Phenom–World BV, Netherlands) with an accelerating voltage of 5 kV was employed for observing the surface morphology of RW and RWC, while the cross sections of different films were observed by a SU8020 scanning electron microscopy (Hitachi, Japan) at an accelerating voltage of 3 kV. The morphology of RW–CNC was characterized by applying a JEM1200EX transmission electron microscope (JEOL, Japan) and the accelerating voltage was 100 kV. The diameter and length distribution of RW-CNC were determined according to a previously published method (Han et al., 2013).

#### Thermal Stability

The thermal stability of different test samples were evaluated by a NETZSCH STA 449 F5 thermogravimetric analyzer and the measuring temperature was programed from 30 to 600°C under a heating rate of 10°C/min. The flow rate of nitrogen was set as 40 mL/min.

#### Mechanical Properties

An Instron 3343 long travel extensometer was applied for measuring the tensile strength and Young’s modulus of different films. The test specimens were pre-cut into stripes (50 mm × 10 mm) and five replicates for each measurement were carried out.

#### Differential Scanning Calorimetry

Thermal behavior of different films was analyzed by applying a differential scanning calorimeter (Q200, TA Instruments Inc., United States). Around 3 mg sample was sealed in an aluminum pan using a sealed empty pan as the reference. In nitrogen atmosphere, the first heating scan was programmed from 20 to 200°C at a heating rate of 10°C/min and held at 200°C for 5 min. Then the temperature was cooled down back to 20°C at the same rate. The second heating scan was carried out afterward from 20 to 200°C at a rate of 10°C/min. The glass transition temperature (*T*_*g*_) was measured from the thermogram of first heating scan, while other thermal parameters including cold crystallization temperature (*T*_*cc*_), melting temperature (*T*_*m*_), and enthalpy of cold crystallization (Δ*H*_*cc*_) and melting (Δ*H*_*m*_) were obtained from the thermographs of second heating scan. Subsequently, the crystallinity of film specimen (*X*_*c*_) was estimated according to an early documented method (Fischer et al., 1973; Sullivan et al., 2015).

#### Light Transmittance

An ultraviolet-visible spectrophotometer (UV-3600 Plus, Shimadzu, Japan) was selected for determination of light transmittance of different film specimens. The wavelength range of incident light is from 200 to 800 nm.

## Results and Discussion

### Chemical Composition of Rubber Wood and Rubber Wood Cellulose

According to the results of composition analysis, RW can be regarded as an ideal source of cellulose, for its high content of glucan (52.8%) based on the dry weight of the starting material (Table 1). Besides fairly low contents of extractives (1.9%) and ash (1.5%), RW contains moderate amount of lignin (20.8% in total) that is summed up by 18.9% of Klason lignin and 1.9% acid soluble lignin. Xylan and mannan are the only two detected types of hemicellulose, whose contents are 18.0 and 2.2%, respectively. The isolation and purification of RWC is quite efficient, since extractives, ash content, and lignin cannot be detected by using traditional quantification methods. The final yield of purified RWC is 34.7% based on the extractive-free raw materials. Furthermore, the purified RWC is highly rich in glucan (91.3%) with a minor portion of xylan (2.2%) and mannan (2.6%), and these values are close to the results published early (Tuerxun et al., 2019).

**TABLE 1:** Chemical composition analysis of RW and purified RWC.

Samples	Extractives (%)	Ash content (%)	Klason lignin (%)	Acid soluble lignin (%)	Glucan (%)	Xylan (%)	Mannan (%)
RW	1.9 ± 0.2	1.5 ± 0.1	18.9 ± 0.4	1.9 ± 0.1	52.8 ± 1.2	18.0 ± 0.5	2.2 ± 0.2
RWC	ND^*a*^	ND^*a*^	ND^*a*^	ND^*a*^	91.3 ± 1.4	2.2 ± 0.1	2.6 ± 0.1

*^*a*^ND: not detectable.*

### FTIR Spectroscopy Analysis of Different Fiber-Based Samples

**FIGURE 2:**
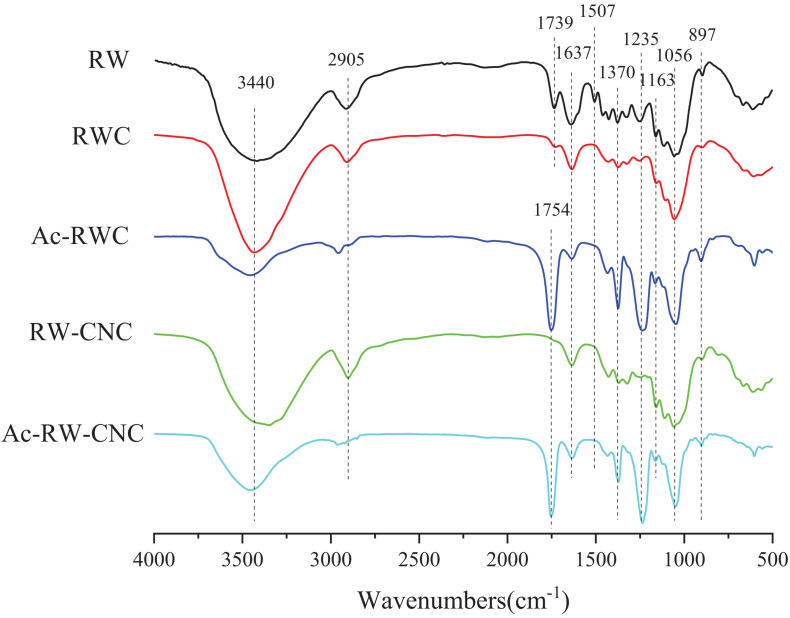
FTIR spectra of RW, RWC, Ac–RWC, RW–CNC, and Ac–RW–CNC.

The FTIR spectra of five different fiber-based samples were demonstrated in Figure 2, the broad adsorption peaks locating around 3,440 cm**^–^**^1^ of all samples are originated from O–H stretching vibration of hydroxyls, whose intensity has been evidently weakened in the cases of Ac–RWC and Ac–RW–CNC owing to their reduced numbers of hydroxyl groups. The characteristic adsorption bands at 2,905 cm**^–^**^1^ stem from C–H stretching vibrations of methyl and methylene groups (Uma Maheswari et al., 2012). The presence of adsorption peak at 1,739 cm**^–^**^1^ is contributed by C = O stretching vibration of original acetyl groups from hemicellulose and carbonyl groups from lignin. However, this band turns out to be diminished, ascribed to the removal of hemicellulose and lignin in RWC. The effective delignification of RWC is also in conformity with the disappearance of aromatic ring skeletal vibration at 1,507 cm**^–^**^1^ (Chen et al., 2011; Ditzel et al., 2017). Moisture content present in sample also result in adsorptions around 1,637 cm**^–^**^1^, which is in line with a previous study published elsewhere (Reddy et al., 2018). Compared with RWC and RW–CNC, the extensively strengthened adsorptions at 1,754 cm**^–^**^1^ and 1,235 cm**^–^**^1^ in their respective acetylated product refer to C = O stretching vibration and C–O–C stretching vibration of acetyl groups. In addition, the increment of –CH_3_ from acetyl groups also accounts for the reinforced intensity of C–H bending vibration at 1,370 cm**^–^**^1^. The characteristic adsorption bands of cellulose locating at 1,163, 1,056, and 897 cm**^–^**^1^ are observed for all test samples (Pappas et al., 2002; Sun et al., 2004).

### X-ray Diffraction Analysis of Different Fiber-Based Samples

**FIGURE 3:**
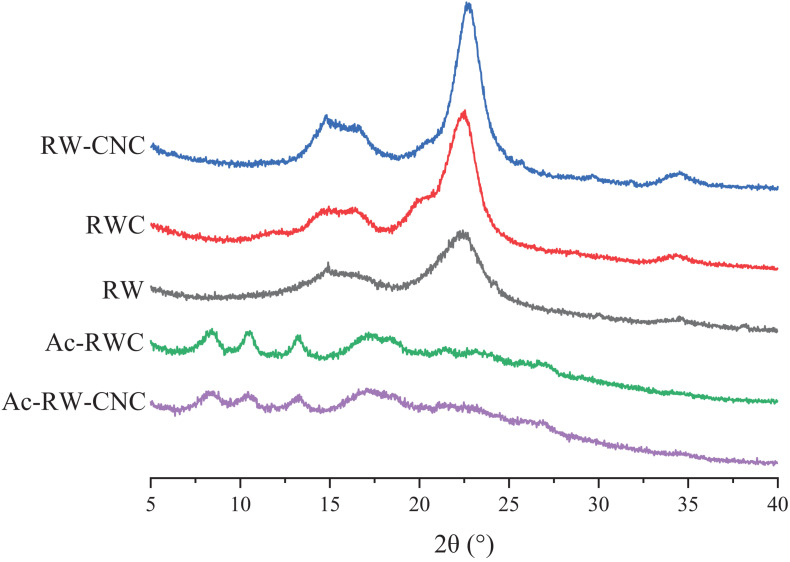
X-ray diffraction patterns of RW, RWC, Ac–RWC, RW–CNC, and Ac–RW–CNC.

The X-ray diffraction patterns of RW, RWC, and RW–CNC are quite similar, in accordance with the pattern of cellulose I (Figure 3). The signals ranging from 13° to 18° are originated from 101 and 10ī planes of cellulose, and the relatively reinforced peak locating around 22° is overlapped by signals of 021 and 002 planes. A solely emerged peak at 34.5° refers to the signal of 040 plane. Although demonstrating analogous X-ray diffraction patterns, these three samples mentioned above still differ in crystallinity. RW, for instance, possesses relatively low crystallinity as 43.0% owing to the existence of moderate amounts of lignin and hemicellulose whose structure are more prone to be amorphous. After effective removal of lignin and majority of hemicellulose, the crystallinity of RWC reaches 65.0%, and this value further climbs up to 74.1% for RW–CNC, since the amorphous domains in RWC had been digested severely during 64 wt% sulfuric acid hydrolysis.

The crystal forms of different fiber samples vary according to their degrees of acetylation. During the early stage of acetylation, the reaction occurred preferentially on the hydroxyl groups within amorphous area, and meanwhile the crystalline region of cellulose remained intact from the attack of acetylation reagents. Therefore, the fiber samples with low substitution degree of acetyl groups still exhibit similar X-ray diffraction pattern as native cellulose. When the reaction continued to proceed, acetylation reagents gradually penetrated into the interior of crystalline sections with an outcome of more interrupted hydrogen bonds that previously played a significant role in maintaining highly crystalline structure of cellulose. Consequently, more distributed and attenuated diffraction peaks have been observed for highly acetylated fiber samples (e.g., Ac–RWC and Ac–RW–CNC) instead of strong characteristic signals, e.g., diffraction peak for 002 plane (around 22°) of purified cellulose product (Figure 3). Based on the findings of a former study, three sharp diffraction peaks from acetylated fiber products, locating at 8.5°, 10.4°, and 13.2° are related to 001 plane, 210 plane, and 310 plane, respectively (He et al., 2008). And a relatively broad peaks around 17.5° are dedicated by 012 plane (He et al., 2008). It is worth mentioning that the higher degree of acetylation results in lower crystallinity of sample.

### Morphology Characterization of Different Fiber-Based Samples

**FIGURE 4:**
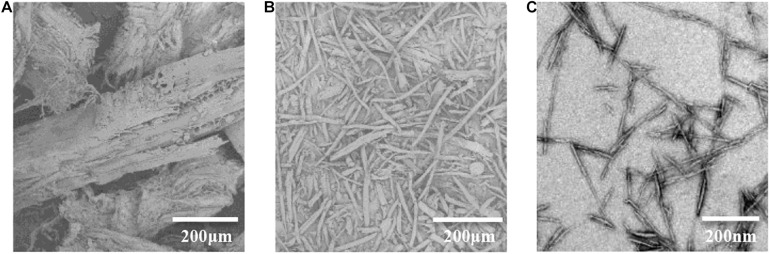
SEM images of RW **(A)** and RWC **(B)** and TEM image of RW–CNC **(C).**

As demonstrated in Figure 4, the extractive-free RW with coarse surface exhibits more opened structure which was beneficial from mechanical disintegration. The presence of massive pores observed on the surface is attributed to the removal of extractives from the raw material (Figure 4A). The surface of RWC turns out to be more smooth and plicated (Figure 4B), which is plausible since lignin and majority of hemicellulose have been efficiently removed during the isolation and purification of RWC. RW–CNC was successfully prepared from RWC *via* 64 wt% sulfuric acid hydrolysis followed by sonication treatment. It is worth noting that RW–CNC aqueous suspension is fairly stable, which is in good agreement with its high zeta potential (–51.8 mV). The sulfonic acid groups introduced during sulfuric acid hydrolysis would endow the surface of RW–CNC with negative charges when present in aqueous media and eventually realize a uniformed dispersion in deionized water (Habibi, 2014). The diameter and length distribution of RW–CNC were obtained, based on the statistical analysis of the entire fibers out of three random selected TEM images. As indicated from the results that the diameters of RW–CNC is mainly in the range of 4–10 nm and the average value is 7.4 ± 2.7 nm (Figure 4C and Supplementary Figure 1A). Furthermore, over 93% of RW–CNCs are scaled in length lower than 400 nm (Supplementary Figure 1B).

### Thermal Stability of Different Fiber-Based Samples

**FIGURE 5:**
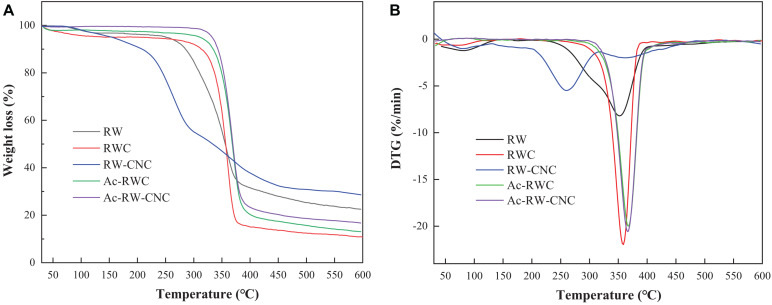
TGA curves **(A)** and DTG curves **(B)** of different fiber-based samples.

Thermogravimetric analysis (TGA) and derivative thermogravimetric analysis (DTG) were conducted to evaluate the thermal stabilities of different fiber-based samples (Figure 5A). The initial stages of weight loss (from 50 to 130°C) for all test samples are explained by the removal of free and bound water from the samples (Wang P. et al., 2020). Later, as for RW, two major decomposition stages are observed (Figure 5B). The first one (from 230 to 320°C) is attributed to the degradation of hemicellulose, since more branched molecular structure renders hemicellulose with more thermally vulnerable in comparison with lignin and cellulose (Shebani et al., 2008). Thermal depolymerization of lignin and cellulose occurred afterward during the second stage ranging from 320 to 410°C and this section is also covering a relatively narrow decomposition stage of RWC. This experimental finding mentioned above is plausible, since RWC is enriched in cellulose with only a minor portion of hemicellulose. Notably, the maximum decomposition temperature (*T*_*max*_) of RWC (359°C) is slightly higher than that of RW (352°C) due to the higher crystallinity of RWC. It is necessary to point out that RW–CNC is the first specimen to decompose at an onset decomposition temperature (*T*_*onset*_) of 200°C, nearly 100°C lower than that of RWC, though its crystallinity ranks the highest out of all test samples. The possible explanation for this evident drop of *T*_*onset*_ can be suggested as follows. Firstly, high crystallinity cannot ensure enhanced thermal stability especially when the sample size belongs to nanoscale, which is in good agreement with our early findings (Wu et al., 2019). Secondly, heat-labile sulfate esters formed on the surface of RW–CNC not only interrupt the linkages of intermolecular hydrogen bonds among RW–CNCs, but also turn out to be more vulnerable during TGA (Tuerxun et al., 2019).

It is worth noting that the thermal stability of RW fibers can be effectively improved by means of acetylation as shown in Figure 5B. The TGA and DTG curves of Ac–RWC and Ac–RW–CNC are fairly consistent, whose primary weight loss takes place from 280 to 430°C with the *T*_*max*_ close to 370°C, even higher than that of RWC (359°C). Compared with sulfate esters and hydroxyl groups, acetyl groups are proved to be more favorable for reinforcement of thermal stability (Wan Daud and Djuned, 2015; Leite et al., 2016).

### Mechanical Properties of Different Films

**FIGURE 6:**
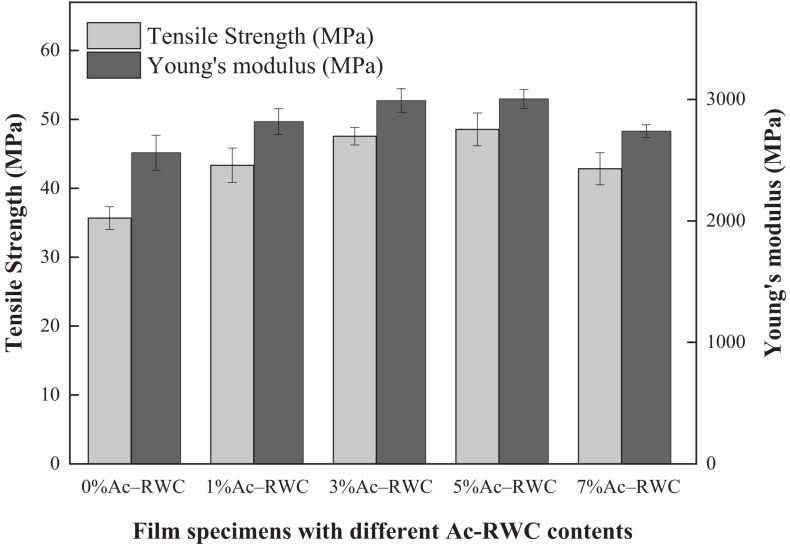
Tensile strength and Young’s modulus of Ac–RWC/PLA composite films.

The mechanical properties of two sets of different films were determined in terms of tensile strength and Young’s modulus. As shown in Figure 6, the addition of Ac–RWC evidently improves the tensile strength of the composite films, compared with pure PLA film (35.7 MPa). When the addition of Ac–RWC reaches 5%, the value of tensile strength is as high as 48.6 MPa. However, this tendency of increment does not persist and the value starts to drop as the addition of Ac–RWC exceeds 5%. The hydrophobic acetyl groups existing on the surface of Ac–RWC strengthen the compatibility between acetylated fibers and PLA matrix. However, the enhanced interactions between filler and matrix would be effected only if the acetylated products could be uniformly distributed in the matrix. Excess involvement of fillers would inevitably lead to local aggregation and eventually weaken the intermolecular forces (Iwatake et al., 2008; Abdulkhani et al., 2014; Sung et al., 2017; Niu et al., 2018). Similar variation trend is also observed when dealing with the values of Young’s modulus. Its maximum value (3,004 MPa) is achieved with 5% addition of Ac–RWC, higher than that of pure PLA film (2,562 MPa).

**FIGURE 7:**
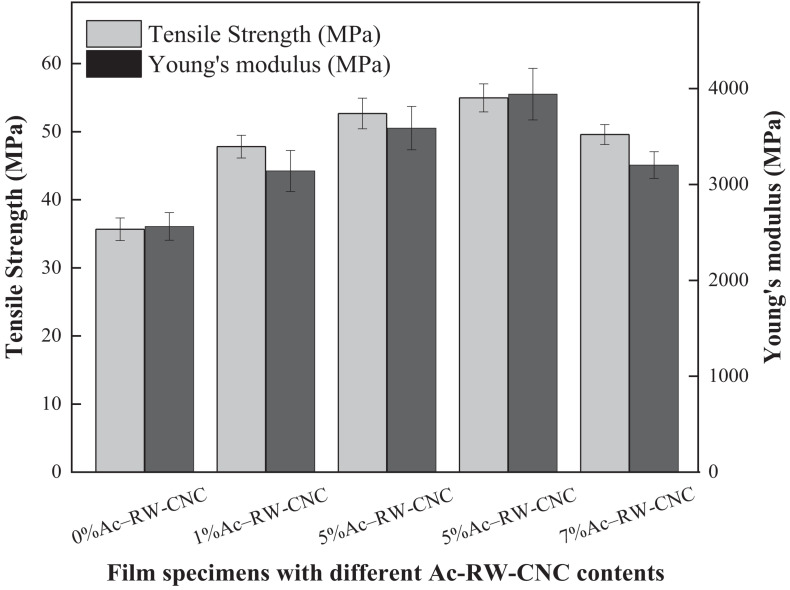
Tensile strength and Young’s modulus of Ac–RW–CNC/PLA composite films.

Although an overall enhancement of mechanical properties has already been obtained for Ac–RWC/PLA composite films, potential means for further improvement can also be attempted by minimizing the filler size (e.g., application of Ac–RW–CNC) for realizing a much more uniformed distribution of filler within PLA matrix. As verified from Figure 7 that, a minor addition of 1% Ac–RW-CNC results in comparable tensile strength and Young’s modulus as those acquired by 5%Ac–RWC/PLA composite film. The tendency of the increments does not cease until the addition of Ac–RW–CNC reaches 5% based on the dry weight of composite film, and the highest values for tensile strength and Young’s modulus are 55.0 and 3,942 MPa, respectively. Similarly as mentioned before, excess addition of filler once again brings about decreased values of mechanical properties due to the aggregation of the fillers, which is well reflected from the results obtained for 7%Ac–RW–CNC/PLA.

### Morphology of Different Films

**FIGURE 8:**
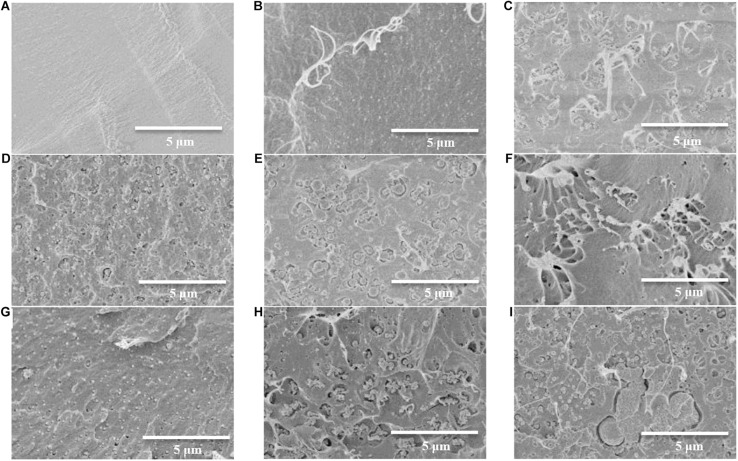
SEM images of the fracture surface of PLA and PLA-based composite films. **(A)** Pure PLA film; **(B)** 1%Ac–RWC/PLA composite film; **(C)** 3%Ac–RWC/PLA composite film; **(D)** 5%Ac–RWC/PLA composite film; **(E)** 7%Ac–RWC/PLA composite film; **(F)** 1%Ac–RW–CNC/PLA composite film; **(G)** 3%Ac–RW–CNC/PLA composite film; **(H)** 5%Ac–RW–CNC/PLA composite film; **(I)** 7%Ac–RW–CNC/PLA composite film.

Compared with the smooth fracture surface of pure PLA film (Figure 8A), the composite films exhibit different degree of roughness (Figures 8B–I). When the addition of reinforcing filler is low, the well distributed fibers would firmly adhere to matrix chains, namely a better interfacial adhesion had been promoted between fillers and PLA matrix. During the fracture process of the film, pulling out the originally firmly embedded fibers would inevitably stretch the surrounding matrix from the fracture surface owing to the strong interfacial adhesion mentioned above (Figures 8B,F) and this phenomenon is more distinct for lower addition of fillers. Besides, the better mechanical properties of 5%Ac–RWC/PLA and 5%Ac–RW–CNC/PLA composite films are also correlative to their higher amounts of evenly distributed fillers (Figures 8D,H). Once the filler dosage exceeds 7%, the aggregation of the fillers could be clearly observed in Figures 8E,I.

### Thermal Stability of Different Films

The TGA and DTG curves of two sets of composite films, designated as Ac–RWC/PLA and Ac–RW–CNC/PLA, were outlined in Supplementary Figures 2, 3. To improve the compatibility of hydrophilic RWC or RW–CNC in hydrophobic PLA matrix, acetylation had been applied in advance. However, the enhanced interfacial adhesion between filler and matrix has been acquired at a cost of sacrificing filler’s high crystallinity due to acetylation. It is widely recognized that the highly crystalline filler can be served as a favorable additive for upgrading the integral thermal stability of the composite films (Xiao et al., 2016). Therefore, the ultimate thermal stability of composite films benefits both from the strengthened interfacial interactions between acetylated-fillers and matrix, and from the remaining crystallinity of the fillers.

In general, the Ac–RWC reinforced films demonstrate similar thermal decomposition behaviors in comparison with pure PLA film (Lizundia et al., 2016). Although the difference is not evident, a series of Ac–RWC/PLA composite films with filler dosage ranging from 1 to 7% are relatively more thermal stable than pure PLA film (Dong et al., 2017; Supplementary Figure 2A). When the filler could be effectively dispersed in PLA matrix, namely the addition of Ac–RWC 5%, the *T*_*max*_ value is observed for a continuous shifting to higher temperature (Supplementary Figure 2B). A sudden shift to a lower *T*_*max*_ occurs, however, when the filler was supplied as 7% based on the dry weight of the film. It could also be explained by the weakened interfacial adhesion arising from the aggregation of fillers. With respect to a set of Ac–RW–CNC/PLA composite films, their thermal stabilities are slightly lower than that of pure PLA film within the temperature difference of roughly 6°C in comparison of individual *T*_*max*_ (Supplementary Figure 3). Relatively lower thermal stability of Ac–RW–CNC with nano-scaled sample size also accounts for the phenomenon observed above.

### Crystallization Behavior of Different Films

**FIGURE 9:**
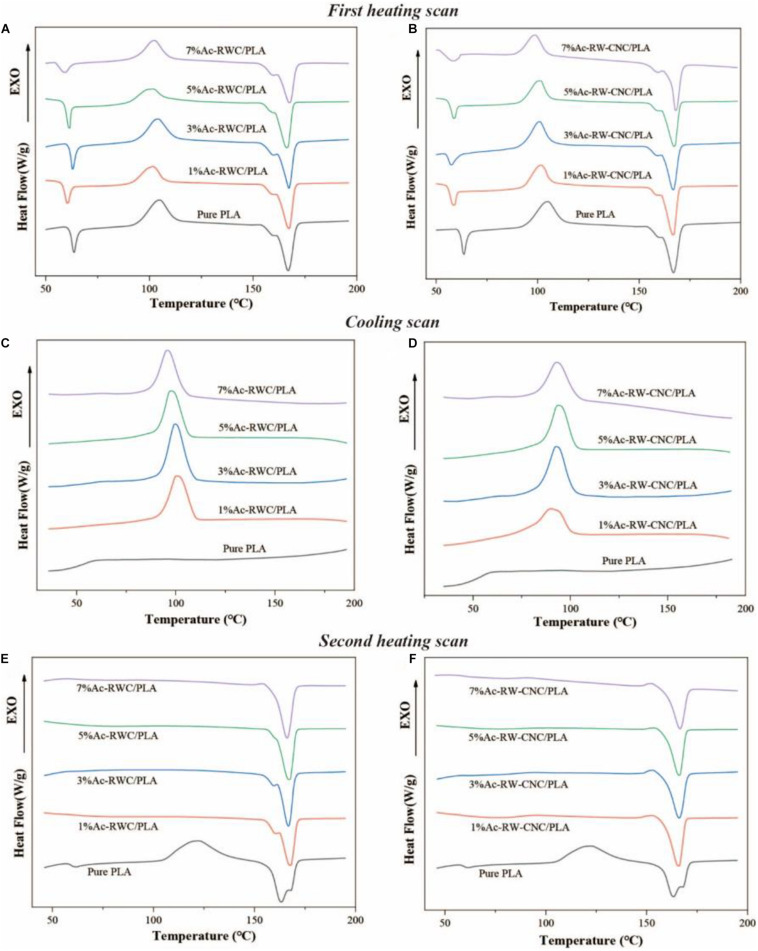
DSC thermograms of PLA and PLA-based composite films. **(A)** The first heating scan of Ac-RWC/PLA composite films. **(B)** The first heating scan of Ac-RW-CNC/PLA composite films. **(C)** The cooling scan of Ac-RWC/PLA composite films. **(D)** The cooling scan of Ac-RW-CNC/PLA composite films. **(E)** The second heating scan of Ac-RWC/PLA composite films. **(F)** The second heating scan of Ac-RW-CNC/PLA composite films.

Compared with other semi-crystalline polymers, PLA has relatively poor thermal stability. For instance, its glass transition temperature is only around 60°C (Figure 9A). A currently prevailing strategy to overcome this drawback is to increase its crystallinity by introduction of nucleating agents (Ghasemi et al., 2012; Kamal and Khoshkava, 2015), since higher crystallinity of PVA-based composite would positively influence its thermal resistance as well as the mechanical properties. As efficient nucleating agents, several species of nanoparticles (e.g., clay and nanotubes) have been previously documented to vary the crystallinity behavior of semi-crystalline polymers by acting as heterogeneous nucleation sites (Ghasemi et al., 2012). Here, in this study, the possibility of Ac–RWC and Ac–RW–CNC to be served as potential candidates for nucleating agents were attempted and their effects on crystallinity behavior of PLA-based composite films had also been investigated.

The crystallization behavior of all specimens during first heating, cooling, and second heating scans are depicted in Figure 9 and their corresponding thermal parameters are summarized in Supplementary Table 1. The DSC thermographs of first heating scan for all test films are quite similar with each other (Figures 9A,B), and the slightly decreased *T*_*g*_ values of composite films accounts for lower influence of fillers on mobility of PLA chains (Sung et al., 2017). During film preparation, rapid evaporation of dichloromethane resulted in sudden cooling of the temperature, which eventually restricted the PLA chains from fully self-adjusted into crystalline structures (Sullivan et al., 2015). Therefore, the exothermic peaks for cold crystallization slightly over 100°C have been observed for all specimens (Figures 9A,B). Compared with pure PLA film, the crystallization behavior of composite films vary significantly throughout the cooling process by emerging evident exothermic peaks of melt crystallization close to 100°C (Figures 9C,D). This phenomenon directly verifies the capacity of nucleation by Ac–RWC and Ac–RW–CNC in composite films under the selected cooling rate (10°C/min), whereas effective crystal nucleus are hardly to be formed within a homogenous system (pure PLA film). Ascribed to the existence of preformed crystalline structure from an early cooling scan, the phenomenon of cold crystallization does not occur during the second heating scan, the situation, however, is opposite respect to pure PLA film (Figures 9E,F). In addition, the *T*_*cc*_ value of PLA film is shifted to higher temperature (around 122°C) in the second heating scan after removal of thermal history.

As shown in Supplementary Table 1, the crystallinity of pure PLA film (4.3%) is much lower than that of composite film, even if the addition of reinforcing filler is only 1%. Generally, the crystallinity of Ac–RW–CNC/PLA composite films is higher than that of Ac-RWC/PLA composite films, and the maximum value is up to 47.6% for 3% Ac-RW-CNC/PLA. It is worth mentioning that the double melting peak observed for pure PLA film during second heating (Figures 9E,F) is indicated by either polymorphism or melt recrystallization (Fortunati et al., 2012; Sullivan et al., 2015).

### Light Transmittance of Different Films

The light transmittance of PLA and PLA-based composite films has been measured throughout a UV-vis range from 200 to 800 nm (Supplementary Figure 4). Theoretically, film transparency is largely depending on the micro-structure of the films (Kim, 2008; Lizundia et al., 2016; Huang et al., 2020). Compared with visible light (400–800 nm), UV light (200–400 nm) is more readily to be absorbed by the test films and the transmittance rate gradually reaches a plateau when the wavelength of incident light exceeds 400 nm. For instance, nearly 93% of visible light could transit pure PLA film and a minor addition (5%) of Ac–RWC or Ac–RW–CNC does not impact the light transmittance to a large extent. 7%Ac–RWC/PLA and 7%Ac–RW–CNC/PLA are the two exceptions, whose light transmittance is dropped to around 85% or even less due to the light scattering and refraction resulted from filler aggregation (Hu and Wang, 2016).

## Conclusion

Rubber wood cellulose had been effectively isolated, processed, and surface modified to acetylated cellulose and acetylated cellulose nanocrystals, both of which could be served as promising reinforcing fillers for fabrication of PLA-based composite films owing to their different structural characteristics as well as the improved compatibility with PLA matrix. By means of spin coating technique, the acetylated cellulose reinforced PLA composite films possess higher thermal stability, while the acetylated cellulose nanocrystals reinforced PLA composite films are more superior in mechanical properties and the degree of crystallinity.

## Data Availability Statement

The raw data supporting the conclusions of this article will be made available by the authors, without undue reservation.

## Author Contributions

ZO and XD proposed supporting the conclusions of this article will be made available by the authors, without undue rthe idea. ZO, XR, and QZ performed most of the experiments. HY and CH helped to revise the manuscript. XD supervised the whole work and wrote the manuscript. All authors contributed to the article and approved the submitted version.

## Conflict of Interest

The authors declare that the research was conducted in the absence of any commercial or financial relationships that could be construed as a potential conflict of interest.
