# Supplementary material for: Cellulose Isolated From Waste Rubber Wood and Its Application in PLA Based Composite Films
Source: Front Bioeng Biotechnol. 2021 Mar 31;9:666399. doi: 10.3389/fbioe.2021.666399 (PMC8044414; doi:10.3389/fbioe.2021.666399)
Supplement: Supplementary file 1 [file Data_Sheet_1.PDF]

Supplementary Material

Supplementary Table 1. Thermal parameters and crystallinity of PLA and PLA-based composite films.

Different films	T_g	T_{cc} (°C)	Δ H_{cc} (J/g)	T_m (°C)	ΔH_m (J/g)	X_c (%)
PLA	63.8±0.7	121.7±0.5	40.04±0.9	163.3±0.6	44.1±0.3	4.3±0.7
1%Ac-RWC/PLA	60.3±0.9	—	—	167.7±0.2	37.2±0.7	40.4±0.8
3%Ac-RWC/PLA	62.9±0.3	—	—	166.8±0.2	39.5±0.8	43.8±0.9
5%Ac-RWC/PLA	61.5±0.8	—	—	167.0±0.5	36.2±1.2	40.9±1.4
7%Ac-RWC/PLA	59.3±0.2	—	—	166.1±1.3	38.9±0.4	45.0±0.5
1%Ac-RW-CNC/PLA	58.4±0.8	—	—	165.6±0.4	42.9±0.6	46.6±0.7
3%Ac-RW-CNC/PLA	57.5±0.5	—	—	166.1±0.9	43.0±0.3	47.6±0.4
5%Ac-RW-CNC/PLA	58.5±0.2	—	—	165.9±1.1	39.7±0.6	44.9±0.7
7%Ac-RW-CNC/PLA	58.8±0.6	—	—	166.4±0.8	40.0±1.3	46.3±1.4

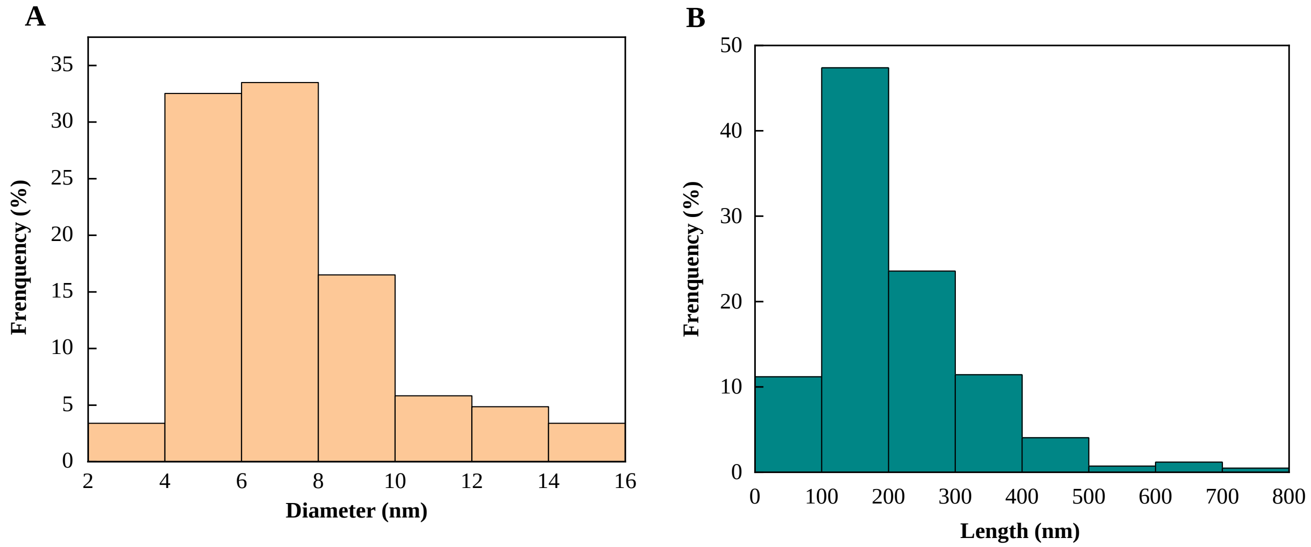

Supplementary Figure 1. The diameter (A) and length (B) distribution of RW–CNC.

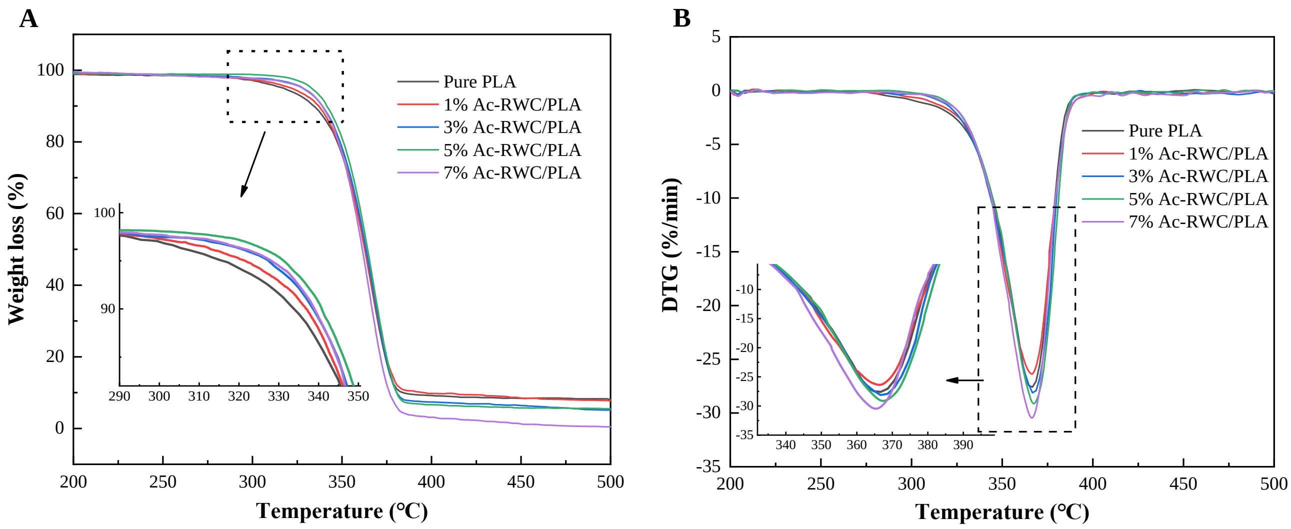

Supplementary Figure 2. TGA (A) and DTG (B) curves of Ac–RWC reinforced PLA composite films.

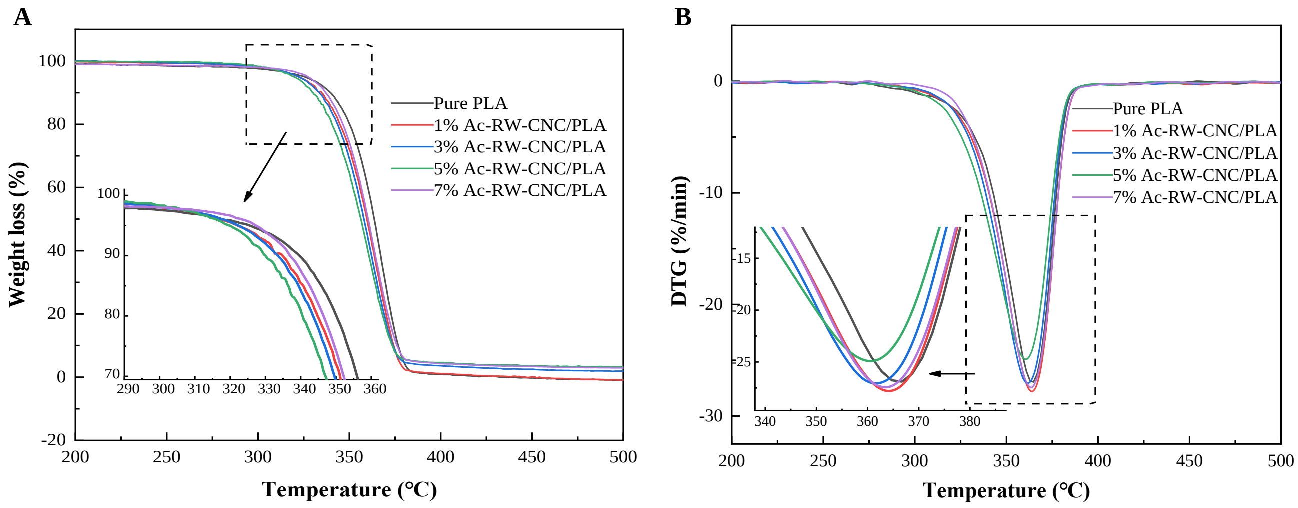

Supplementary Figure 3. TGA (A) and DTG (B) curves of Ac–RW–CNC reinforced PLA composite films.

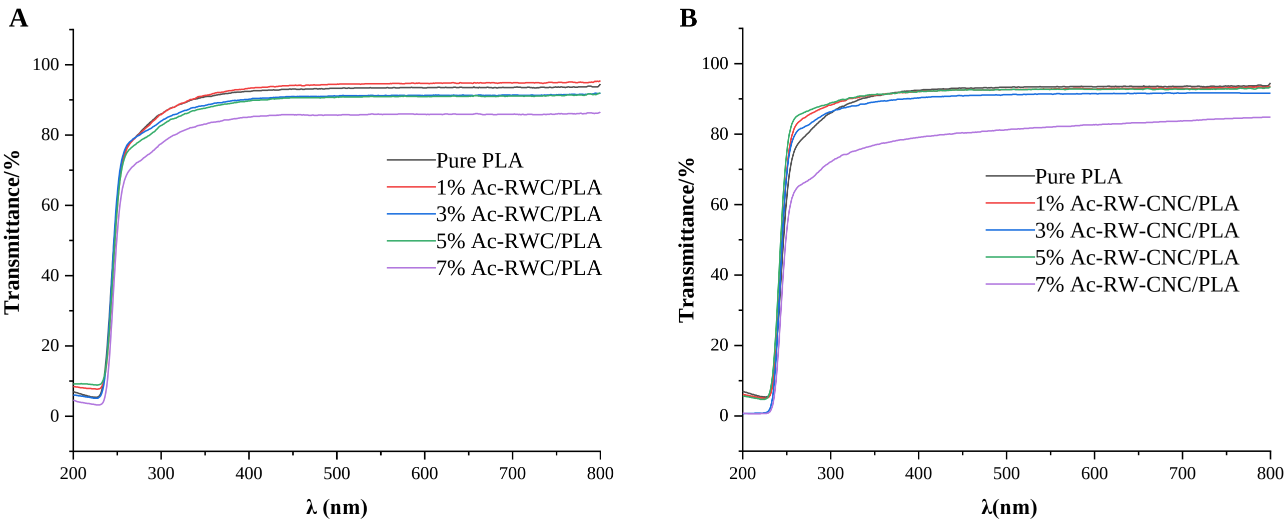

Supplementary Figure 4. Light transmittance of PLA and PLA-based composite films. (A) a series of Ac–RWC/PLA composite films; (B) a series of Ac–RW–CNC/PLA composite films.
